# Striatal connectivity changes following gambling wins and near-misses: Associations with gambling severity

**DOI:** 10.1016/j.nicl.2014.06.008

**Published:** 2014-07-03

**Authors:** Ruth J. van Holst, Henry W. Chase, Luke Clark

**Affiliations:** a Department of Psychology, University of Cambridge, Downing Street, Cambridge CB2 3EB, United Kingdom; b Donders Institute for Cognition, Brain and Behaviour, Radboud University, PO Box 9101, Nijmegen 6500 HB, The Netherlands; c Translational Neuroscience Programme, University of Pittsburgh Medical Center, 3811 O'Hara Street, BST W1654, Pittsburgh, PA 15213, United States; d Centre for Gambling Research at UBC, Department of Psychology, University of British Columbia, 2136 West Mall, Vancouver, B.C., V6T 1Z4, Canada

**Keywords:** Gambling, Connectivity, fMRI, Reward, Near-miss, Addiction

## Abstract

Frontostriatal circuitry is implicated in the cognitive distortions associated with gambling behaviour. ‘Near-miss’ events, where unsuccessful outcomes are proximal to a jackpot win, recruit overlapping neural circuitry with actual monetary wins. Personal control over a gamble (e.g., via choice) is also known to increase confidence in one's chances of winning (the ‘illusion of control’).

Using psychophysiological interaction (PPI) analyses, we examined changes in functional connectivity as regular gamblers and non-gambling participants played a slot-machine game that delivered wins, near-misses and full-misses, and manipulated personal control. We focussed on connectivity with striatal seed regions, and associations with gambling severity, using voxel-wise regression.

For the interaction term of near-misses (versus full-misses) by personal choice (participant-chosen versus computer-chosen), ventral striatal connectivity with the insula, bilaterally, was positively correlated with gambling severity. In addition, some effects for the contrast of wins compared to all non-wins were observed at an uncorrected (*p* < .001) threshold: there was an overall increase in connectivity between the striatal seeds and left orbitofrontal cortex and posterior insula, and a negative correlation for gambling severity with the connectivity between the right ventral striatal seed and left anterior cingulate cortex.

These findings corroborate the ‘non-categorical’ nature of reward processing in gambling: near-misses and full-misses are objectively identical outcomes that are processed differentially. Ventral striatal connectivity with the insula correlated positively with gambling severity in the illusion of control contrast, which could be a risk factor for the cognitive distortions and loss-chasing that are characteristic of problem gambling.

## 1 Introduction

Good decision making is partly dependent on the ability to accurately evaluate the outcomes of decisions, and past research using functional magnetic resonance imaging (fMRI) has implicated a network of regions in reward processing and incentive-based learning, including the orbitofrontal cortex (OFC), amygdala, insula, and ventral striatum/nucleus accumbens (Elliott et al., 2000; Schultz et al., 2000; Knutson et al., 2001; Pagnoni et al., 2002; Elliott et al., 2003; Haber et al., 2006). Abnormal recruitment of these regions is associated with pathological risk taking and addictive behaviours, including problem gambling (e.g., Verdejo-Garcia and Bechara, 2009; van Holst et al., 2010). Recent connectivity research has enabled a further characterization of this circuitry, showing that during monetary gains and losses, functional connectivity increases between the ventral striatum and the medial prefrontal cortex (e.g., Camara et al., 2008; Harsay et al., 2011; Cohen et al., 2012; Park et al., 2012; van den Bos et al., 2012). For example, Park et al., showed that effective coding of prediction errors (i.e. the difference between the obtained and the expected outcome) was signalled by changes in connectivity between the striatum and reward-sensitive regions in the midbrain and medial prefrontal cortex, with individual differences in connective strength being further predictive of behavioural responses (Park et al., 2010; Harsay et al., 2011; Wallace et al., 2011).

Gambling games are an ideal probe to investigate this circuitry, because of the ability to maximize outcome uncertainty and elicit anticipation, via real monetary wagers and outcomes (Clark, 2010; Kishida and Montague, 2012). Work with realistic gambling games has begun to highlight a number of novel features that modulate reward responsivity. One example is the ‘near-miss effect’, when an unsuccessful outcome is proximal to a designated win, such as when two cherries are displayed on a three-reel slot machine. Near-misses are reported as subjectively unpleasant but enhance motivations to gamble (Clark et al., 2009), and manipulating the frequency of near-misses influences gambling persistence (Kassinove and Schare, 2001; Cote et al., 2003). Using neuroimaging, near-misses were shown to recruit parts of the brain reward system that overlapped with responses to the actual wins, in the ventral striatum and anterior insula (Clark et al., 2009). In a neuropsychological study, damage to the insula was also associated with a diminished sensitivity to near-misses, as well as the ‘gambler's fallacy’ (Clark et al., 2014). In regular gamblers, the level of problematic gambling (on the South Oaks Gambling Screen) predicted a greater response to near-misses in a midbrain region proximal to substantia nigra (Chase and Clark, 2010), implying that these near-miss effects may be potentiated in problem gamblers (Chase and Clark, 2010; Habib and Dixon, 2010).

Another feature that has been shown to influence gambling behaviour is the ‘illusion of control’. It is frequently observed that gamblers have an inflated confidence when given the opportunity to choose their lottery ticket or throw dice or roulette ball themselves, compared to the condition where the action is performed by another (Langer, 1975; Stefan and David, 2013). Of course, in a game of chance, personal control carries no objective benefit. The presence of personal control may be a core factor in causing the gambler to mistake a game of chance for a game with some skill component. In a previous study using a two-reel slot machine task, the neuronal correlates of this ‘illusion of control’ were assessed by manipulating whether the participant or the computer selected the ‘play icon’ prior to each reel spin (Clark et al., 2009; Chase and Clark, 2010). An interaction between personal control and near-miss outcomes was expressed in rostral anterior cingulate cortex (ACC) (Clark et al., 2009), and signal in this area was attenuated in regular gamblers (Chase and Clark, 2010).

As a clinical entity, pathological gambling has been reliably associated with abnormal activation patterns in this brain reward circuitry on gambling-like games (Reuter et al., 2005; Tanabe et al., 2007; de Ruiter et al., 2009; Balodis et al., 2012; Miedl et al., 2012; van Holst et al., 2012a). However, the direction of the reported abnormal responses in the reward system has been far from consistent (van Holst et al., 2012b), with some studies reporting diminished win-related activity in pathological gamblers (Reuter et al., 2005; Tanabe et al., 2007; de Ruiter et al., 2009; Balodis et al., 2012), and others describing hyper-activity in the same areas (Miedl et al., 2010; Miedl et al., 2012; van Holst et al., 2012a). Similar discrepant findings are observed in drug-addicted cohorts (for reviews see; Hommer et al., 2011; Limbrick-Oldfield et al., 2013). Analysis of functional connectivity changes may help clarify these disruptions in underlying processing, in order to resolve these seemingly contradictory findings. For example, individuals with alcohol dependence were found to have intact striatal processing of reward value, but a behavioural impairment in the subsequently learning from these signals, which was further predicted by functional connectivity between the striatum and prefrontal cortex (Park et al., 2010). Thus, while task-related increases or decreases seem to be sensitive to minor changes in tasks and/or analysis models, changes in functional connectivity may be more reliable as they indicate network-level integrity.

The present study sought to examine the neural correlates of gambling near-misses and illusion of control, focussing on changes in fMRI connectivity. We had two aims: the first was to investigate the overall changes in functional connectivity following different gambling outcomes, combining the non-gambling participants from Clark et al. (2009) with the regular gamblers from Chase and Clark (2010). The second objective was to characterize the associations between connectivity changes and gambling severity, treating SOGS score as a continuous variable indexing problem gambling. We used a generalized psychophysiological interaction analysis (gPPI: McLaren et al., 2012) in order to model changes in connectivity across different task conditions: 1) wins versus all non-wins, 2) near-misses versus full-misses, and 3) the interaction of near-misses (versus full-misses) by personal choice (participant-chosen versus computer-chosen trials). We selected seed regions in the striatum from the GLM contrast of wins minus all non-wins, given that striatal foci are robustly activated in reward-related processing and decisional tasks (Liu et al., 2011), and the striatum is anatomically situated as a ‘hub’ with reciprocal connections with other reward areas (Haber and Knutson, 2010).

## 2 Methods

### 2.1 Subjects

Non-regular gamblers (*n* = 15, 6 females) and regular gamblers (*n* = 20, 2 females) were recruited via advertisement as described in previous studies (Clark et al., 2009; Chase and Clark, 2010). Regular gamblers were defined as weekly gamblers. As the two studies were designed separately, the groups were not intended to be matched for demographic factors. All participants completed the South Oaks Gambling Screen (SOGS; Lesieur and Blume, 1987), a 16-item self-report scale assessing core symptoms and negative consequences of gambling (e.g., loss chasing, borrowing money, lying about gambling, family conflict). The SOGS was therefore used to investigate individual differences in gambling problems in relation to connectivity patterns. Subjects reported minimal to extensive involvement in gambling, indexed by scores on the South Oaks Gambling Screen of 0–19 (mean 1.91, SD 8.50, scores >5 indicate probable pathological gambling). Thirteen of the group met the SOGS threshold of ≥5 for probable Pathological Gambling (comorbidities are reported in Chase and Clark, 2010).

Two subjects were excluded from the analysis due to technical problems, leaving a reported group size of 33: non-regular gamblers *n* = 14 (5 females) and regular gamblers *n* = 19 (1 female). Subjects attended an fMRI scanning session at the Wolfson Brain Imaging Centre, Cambridge, U.K. The protocol was approved by the Norfolk & Norwich Research Ethics Committee (COREC 06/Q0101/69) and all volunteers provided written informed consent. Volunteers were reimbursed £40 for participation, with a further task-related bonus of £15. The regular gamblers completed a structured psychiatric interview with a postdoctoral psychologist (Structured Clinical Interview for DSM-IV Axis I Disorders; SCID) (First et al., 1996), reported in Chase and Clark (2010).

### 2.2 Procedure

During the fMRI scan, subjects completed 3 blocks of 60 trials on a slot machine game (Clark et al., 2009; Chase and Clark, 2010). On each trial, two reels were presented, with a horizontal ‘pay-line’. Six icons were displayed on each reel, in the same order. Each trial commenced with a selection phase: on participant-chosen trials with a white screen background, the subject selected the play icon using two buttons to scroll through the shapes, and a third button to confirm selection. On computer-chosen trials with a black screen background, the computer selected the play icon, and the subject was required only to confirm selection with a key press. Following selection (5 s), the right reel spun for a 2.8–6 s anticipation phase, and decelerated to a standstill, beginning the outcome phase (4 s fixed). At the end of each trial, there was an inter-trial interval of variable duration (2–7 s). In the outcome phase, if the right reel stopped on the selected icon (i.e. matching icons were displayed in the pay-line), a £0.50 win was delivered; all other outcomes won nothing. Trials where the right reel stopped one position above or below the pay-line were designated ‘near- misses’. Non-win trials where the reel stopped in one of the three remaining positions (i.e. more than one position from the pay-line) were designated ‘full-misses’. Participant-chosen (*n* = 90) and computer-chosen trials (*n* = 90) were presented in a fixed pseudo-random order, which delivered a fair number of wins (1/6), near-misses (2/6) and full-misses (3/6), and a total profit of £15 that was paid on completion. Subjective ratings were acquired on 1 in 3 trials (at random), using onscreen 21-point visual analogue scales: following selection, subjects rated “How do you rate your chances of winning?” and following the outcome, subjects rated “How much do you want to continue to play the game?”.

Data from the subjective ratings were converted to standardized *z* scores, based on each individual's mean and standard deviation for that rating, to account for the variability in anchoring across subjects. Subjective ratings were analysed using repeated-measures analysis of covariance (ANCOVA). The ANCOVA for the ‘chances of winning’ rating control (participant-chosen, computer-chosen) as a factor and SOGS score as a covariate. For the ‘continue to play’ rating, outcome (3 levels: win, near-miss, full-miss) and control (2 levels: participant-chosen, computer-chosen) were repeated-measures factors, and SOGS score as a covariate.

### 2.3 Imaging procedure

Scanning was performed on a Siemens TimTrio 3 Tesla magnet using a 32 slice axial oblique sequence, with a repetition time of 2 s (TE 30 ms, flip angle 7, voxel size 3.1 × 3.1 × 3.0 mm, matrix size 64 × 64, field of view 201 mm × 201 mm, bandwidth 2232 Hz/Px). At the start of each run, six dummy scans were discarded to allow for equilibrium effects. Each 60 trial EPI run lasted a maximum of 630 repetitions (21 min), but was terminated early on block completion. A high resolution T1-weighted three-dimensional magnetization-prepared rapid acquisition gradient-echo sequence (MP-RAGE) structural image was also acquired for use in spatial normalization of the EPI series.

### 2.4 Analyses

#### 2.4.1 Standard GLM analyses

The fMRI data analysis and pre-processing was performed using SPM8. Data preprocessing consisted of slice timing correction, within-subject realignment, spatial normalization, and spatial smoothing using a 10 mm Gaussian kernel. The time series were high pass filtered (128 s). Volumes were normalized to the International Consortium for Brain Mapping (ICBM) templates that approximate to Talairach and Tournoux (1988) space, using a matrix obtained from normalizing each subject's segmented MP-RAGE structural scan onto the ICBM grey and white matter templates. A canonical haemodynamic response function (HRF) was modelled to the onsets of the selection phase, the anticipation phase and the outcome phase on each trial. At the selection onset, two trial types were distinguished: participant-chosen trials and computer-chosen trials. At both anticipation and outcome, eight trial types were distinguished, comprising a 2 (choice: participant-chosen, computer-chosen) by 4 (win, near-miss before the payline, near-miss after the payline, full-miss) factorial design. The design matrix thus comprised 18 (2 + 8 + 8) columns for each of the three sessions (54 task regressors), as well as the movement parameters from realignment which were included as regressors of no interest. The HRF was used a covariate in a general linear model, and a parameter estimate was obtained for each voxel, for each event type, reflecting the strength of covariance between the data and the canonical HRF. Results of these fMRI analyses for each specific group have been reported previously (Clark et al., 2009; Chase and Clark, 2010). We use a whole-brain voxel-wise FWE *p* < 0.05 corrected threshold to report the results of the GLM analyses.

#### 2.4.2 Selection of seed regions

**Fig. 1:**
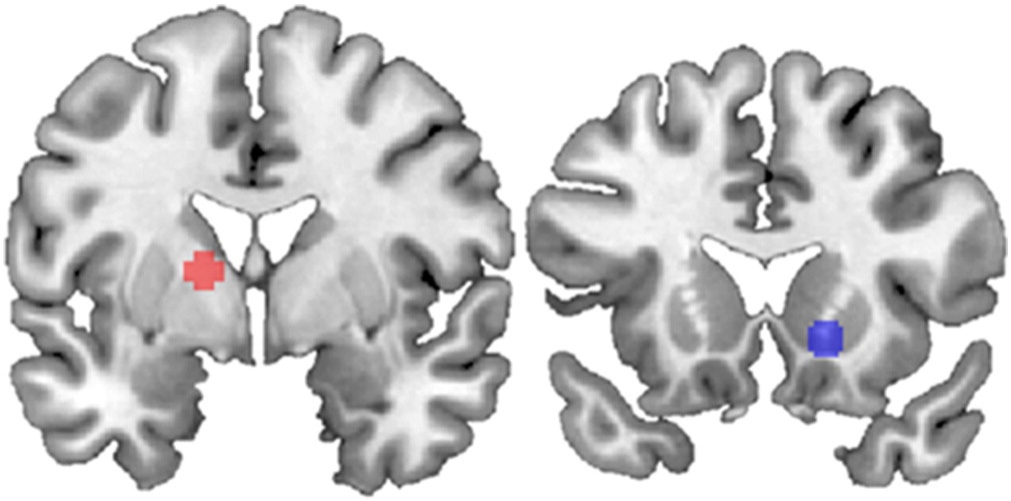
Right and left striatal seeds based on peak activation. The left dorsal medial striatum seed is depicted in red. The right ventral striatum seed is depicted in blue.

Seeds for the gPPI analyses were identified using the GLM contrast for win-related activity (all win–all misses) in the pooled sample of 33 participants, using the FWE *p* < 0.05 corrected threshold. We selected two seed regions defined as 5 mm radius spheres, around the peak striatal voxels on either hemisphere, resulting in a right ventral striatum (*x*, *y*, *z*: 16, 18, –6) seed and a left dorsal medial striatum (*x*, *y*, *z*: –14, 0, 8) seed (see Fig. 1).

#### 2.4.3 Generalized PPI method

Data from the two original studies (Clark et al., 2009; Chase and Clark, 2010) was pre-processed in SPM8, employing the original (and identical) GLM design matrix from those studies. The generalized PPI toolbox (gPPI; http://www.nitrc.org/projects/gppi; McLaren et al., 2012) in SPM8 (Statistical Parametric Mapping, Wellcome Department of Cognitive Neurology, London, UK) was used for connectivity analysis, given that gPPI has the flexibility to accommodate multiple task conditions in the same connectivity model. For each subject, the underlying neuronal activity that produced the physiological activity in the seed region was estimated by deconvolving the BOLD signal (Gitelman et al., 2003) of the 54 task regressors. Then, a region of interest (ROI) analysis (single-subject level) was performed using the general linear model in SPM8 with the 54 PPI regressors, the 54 task regressors, 18 movement parameters and the mean time course in the seed region. For both seed regions, 3 PPI contrasts were created:

1 Wins–all non-wins.
2 Near-misses–full-misses.
3 Interaction effect of (near-miss–full-miss) × (participant-chosen–computer-chosen trials).

**Fig. 2:**
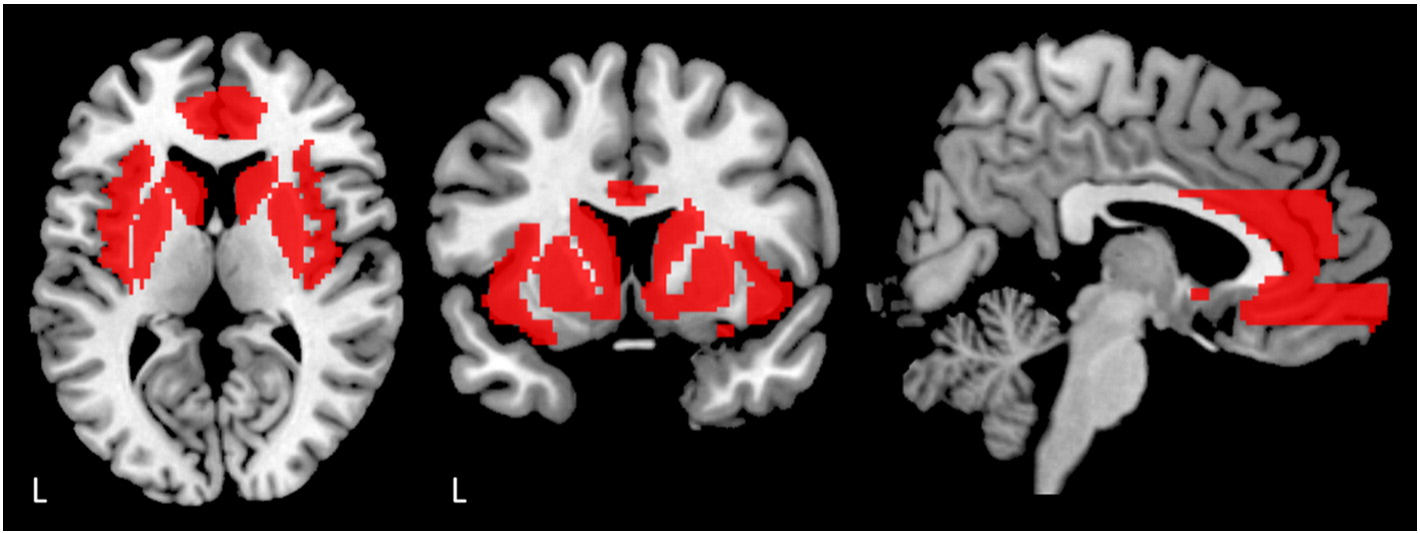
ROI including the bilateral caudate, putamen, insula, anterior cingulate cortex, middle orbitofrontal cortex and medial orbitofrontal cortex was used in the PPI analysis.

These PPI contrast images were then entered into a one-sample *t*-test at the group level, to test for group effects of the three contrasts. In addition, significant connectivity responses were followed up with regressions against gambling severity for each seed. The regression analyses with gambling severity controlled for age and gender by including these variables as regressors in the multiple regression analyses. The ROI was defined anatomically using WFU PickAtlas (based on the AAL atlas), comprising bilateral caudate and putamen, insula, anterior cingulate cortex, middle orbitofrontal cortex and medial orbitofrontal cortex (see Fig. 2). Connectivity analyses were thresholded at voxel-wise *p* < 0.05 FWE corrected threshold with the ROI; subthreshold effects at a voxel-wise *p* < 0.001 uncorrected threshold (cluster threshold of *k* = 5) are also noted as preliminary observations.

## 3 Results

### 3.1 Subjective ratings and gambling severity

The ratings data were analysed using repeated-measures ANCOVA, with SOGS scores included as covariate. The post-selection ratings of “How do you rate your chances of winning?” were significantly higher on participant-chosen trials compared to computer-chosen trials, *F*(1,32) = 20.05, *p* < 0.001, but did not vary as a function of SOGS score, *F*(1,32) = 0.003, *p* < 0.958.

**Fig. 3:**
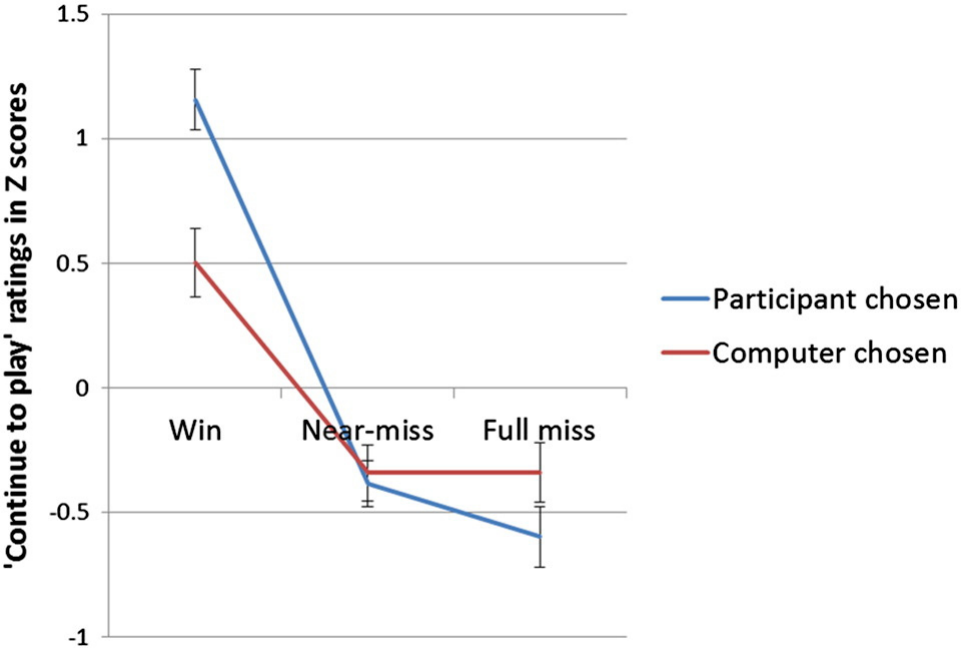
‘Continue to play’ ratings were higher after participant-chosen wins than for computer chosen wins. The bar reflects the standard errors of the mean.

On the post-outcome ratings of “How much do you want to continue to play?”, there was a significant main effect of Outcome, *F*(2,62) = 27.85, *p* < 0.001, driven largely by the winning outcomes (see Fig. 3), and a Choice by Outcome interaction, *F*(2,62) = 13.43, *p* < 0.001. The main effect of Choice was not significant, *F*(1,32) = 2.64, *p* = 0.114. ‘Continue to play’ ratings were higher after participant-selected wins than for computer-selected wins, *t*(32) = 3.51, *p* = 0.001. Participant-chosen near-misses were not significantly different from the participant-chosen full-misses, *t*(32) = 1.493, *p* = 0.145, nor was there a significant difference between computer-chosen near-misses and computer-chosen full-misses, *t*(32) = 0.014, *p* = 0.989. There was a 3-way interaction of the gambling severity (SOGS) term by Outcome by Choice, *F*(2,62) = 6.44, *p* < 0.001, driven by a positive correlation between SOGS score and the ‘continue to play’ rating following computer-chosen wins, *r*_33_ = .37, *p* = 0.032, and a negative correlation between SOGS score and the rating on computer-chosen full-misses, *r*_33_ = −.41, *p* = 0.012. Hence, gambling severity predicted greater motivations to play after computer-selected wins, and lower motivations after computer-selected full-misses. There were no significant correlations between gambling severity and the difference score for near-miss–full miss participant chosen ratings, nor with the difference score for near-miss and full miss computer chosen ratings.

### 3.2 GLM results

The contrast of wins minus all non-win outcomes (whole-brain voxel-wise FWE < 0.05 corrected) showed activations in the bilateral striatum (right: *x*, *y*, *z*: 16, 18, –6, *Z* = 5.90; left: *x*, *y*, *z*: –14, 0, 8, *Z* = 5.78) that were used to identify the two seeds, as well as right thalamus (*x*, *y*, *z*: 2, –16, 2, *Z* = 6.39), left insula (*x*, *y*, *z*: –36, 18, –6, *Z* = 5.29), bilateral ACC (right: *x*, *y*, *z*: 0, 28, –4, *Z* = 4.70; left: *x*, *y*, *z*: –4, 40, 0, *Z* = 5.04), right middle cingulate (*x*, *y*, *z*: 8, –44, 34, *Z* = 4.62) and bilateral cuneus (right: *x*, *y*, *z*: 8, –72, 34, *Z* = 4.63; left: *x*, *y*, *z*: –12, –76, 6, *Z* = 4.62).

The contrast of near-misses minus full-miss outcomes (whole-brain voxel-wise FWE < 0.05 corrected) indicated activity in the right insula (*x*, *y*, *z*: 38, 22, 4, *Z* = 4.76) and right striatum (*x*, *y*, *z*: 14, 6, –2, *Z* = 4.71), as described previously (Clark et al., 2009; see also Shao et al., 2013; Dymond et al., 2014).

There were no significant effects (whole-brain voxel-wise FWE < 0.05 corrected) for the interaction between near-misses and personal control.

### 3.3 Functional connectivity during processing of gambling outcomes

**Fig. 4:**
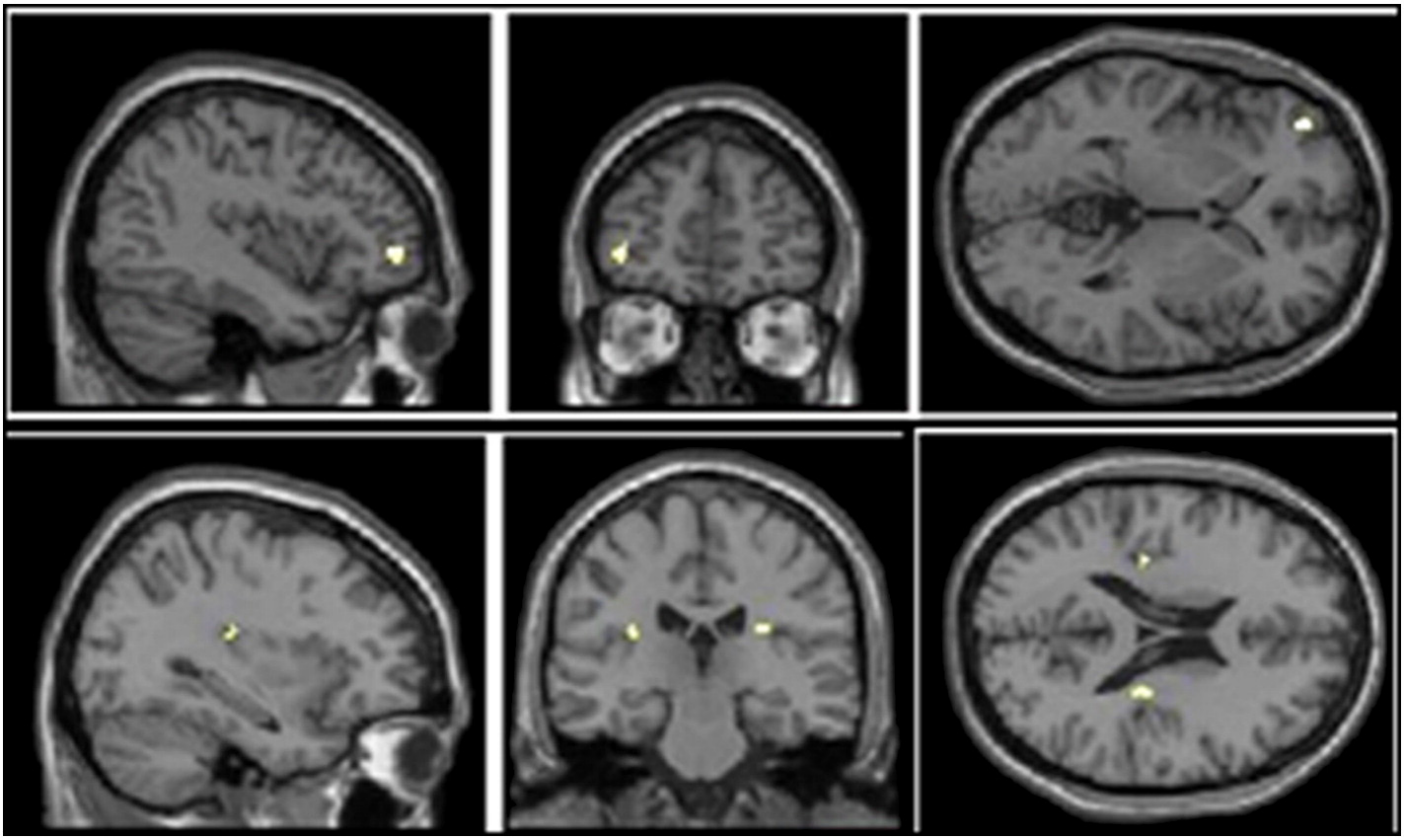
There were increases in connectivity for the left dorsal medial striatum seed in the left orbitofrontal (BA 10, *x*, *y*, *z*: –40, 48, –2, *Z* = 4.16; *k* = 48), and the right anterior cingulate cortex (BA 32, *x*, *y*, *z*: 8, 36, 20, *Z* = 3.62; *k* = 10). For the right ventral striatum seed there was increases in connectivity in the bilateral posterior insula (*x*, *y*, *z*: –32, –24, 20, *Z* = 3.59, *k* = 6).

Following winning outcomes compared to all non-win outcomes, there were subthreshold (i.e. *p* < .001 uncorrected) increases in connectivity for the left dorsal striatum seed in the left orbitofrontal cortex (BA 10, *x*, *y*, *z*: –40, 50, –2, *Z* = 3.62, *k* = 10) and for the right ventral striatum seed in the bilateral posterior insula (left: *x*, *y*, *z*: –32, –24, 20, *Z* = 3.59, *k* = 6, and right: *x*, *y*, *z*: 34, –20, 22, *Z* = 3.46, *k* = 5) (see Fig. 4).

The near-miss minus full-miss contrast, and the interaction contrast for near-misses (versus full-misses) by personal control, both showed no significant modulation of functional connectivity for either seed region.

### 3.4 Effects of gambling severity: functional connectivity

**Fig. 5:**
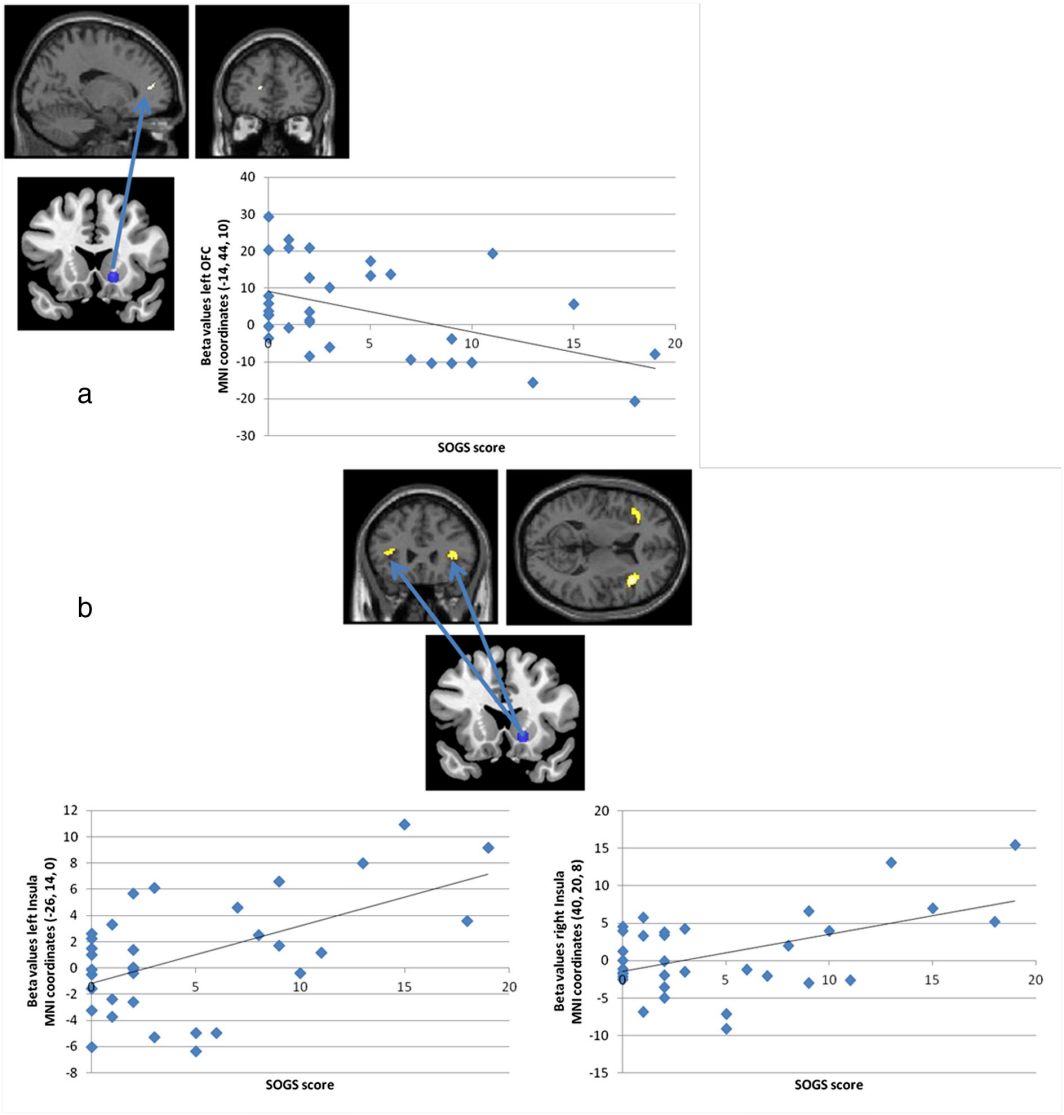
a: Gambling severity was negatively correlated with connectivity between the right ventral striatum seed and the left ACC (*x*, *y*, *z*: –14, 44, 10, *Z* = 3.23, *k* = 6). b: In the interaction of near-misses by personal control contrast, gambling severity positively predicted connectivity between the right ventral striatal seed and the bilateral insula (right: *x*, *y*, *z*: 40, 20, 8, *Z* = 4.08, *k* = 32 and for left: *x*, *y*, *z*: –26, 14, 0, *Z* = 3.60, *k* = 16).

We tested whether the changes in functional connectivity in the win–all non-win, near-miss–full misses, and the near-miss by personal control interaction term were further correlated with gambling severity. For the win–non-win contrast, there was a subthreshold negative correlation between gambling severity and the connectivity between the right ventral striatal seed and the left ACC (*x*, *y*, *z*: –14, 44, 10, *Z* = 3.23, *k* = 6, see Fig. 5a): more severe gambling problems were related to weaker connectivity between the right ventral striatum and the left ACC following wins. There were no associations with severity for the left dorsal striatal seed. For the near-miss–full-miss contrast, there were no significant correlations between functional connectivity and gambling severity for either seed.

For the interaction of near-misses by personal control, gambling severity positively predicted connectivity between the right ventral striatal seed and the bilateral insula (right: *x*, *y*, *z*: 40, 20, 8, *Z* = 4.08; *p* < 0.05 FWE corrected, *k* = 32) with a subthreshold effect for the contralateral region: *x*, *y*, *z*: –26, 14, 0, *Z* = 3.60, *k* = 16, *p* < 0.001 uncorrected, see Fig. 5b. Thus, as gambling severity increased, the connectivity became stronger between the right ventral striatum and insula for self-selected near-misses (compared to full-misses) relative to computer-selected near-misses (compared to full-misses). To decompose this effect, we compared beta values in the right and left insula for the participant-chosen trials (near-misses versus full-misses) and the computer-chosen trials separately. Positive correlations were observed between the beta values for the participant-selected contrast and SOGS scores in the right insula (*r* = 0.451, *p* = 0.008) and left insula (*r* = 0.345, *p* = 0.050). For the computer-selected contrast the correlations with SOGS score were non-significant. Thus, the insula connectivity effect was driven by a positive correlation with gambling severity following participant-chosen outcomes.

## 4 Discussion

This study investigated the patterns of functional connectivity following win and near-miss outcomes on a slot-machine game. We examined how functional connectivity following near-misses was modulated by personal control; compared trials where either the participant or the computer selected the play icon, putatively reflecting the ‘illusion of control’ (Langer, 1975). We also examined the relationships between the connectivity measures and gambling severity on an established self-reported symptom scale, the South Oaks Gambling Screen. Many of our results did not meet FWE (*p* < .05) significance but these subthreshold effects were nevertheless consistent with past research, showing that winning outcomes increased connective strength between a seed in the left dorsal (medial) striatum and the OFC, and between a right ventral striatum seed and posterior insula (Peters and Buchel, 2010; Ballard et al., 2011; Cohen et al., 2012; Park et al., 2012). In distinguishing the two types of non-win outcomes−near-misses and full-misses−no connectivity changes were observed following near-misses for either striatal seed.

For win outcomes, gambling severity negatively predicted connectivity between the right ventral striatum and the ACC. Thus, more severe gamblers display weakened win-related connectivity between established components of the reward network. These connectivity results are consistent with our previous findings indicating a weaker response in regular gamblers (a group that included some problem gamblers) to monetary wins in several reward-sensitive regions including the striatum and rostral anterior cingulate cortex (Chase and Clark, 2010) (although we note that the connectivity analysis is itself based on an extended sample from the GLM analysis). Attenuated responses in pathological gamblers to monetary outcomes have also been reported by previous case control studies (Reuter et al., 2005; Balodis et al., 2012). These results have led to the hypothesis that pathological gambling suffer from an overall diminished reward sensitivity, reminiscent of findings in individuals with drug dependence (Beck et al., 2009; Bustamante et al., 2013; Patel et al., 2013). Our current study extends previous data by indicating that gambling severity is associated with less connectivity between reward-sensitive areas. Our findings complement the study by (Park et al., 2010), which reported an attenuated frontal-striatal connectivity following different monetary outcomes in alcohol dependent patients. They postulated that “enhanced connectivity during reward contexts provides a mechanism that enables reinforcement of the current action in the dlPFC by striatal reward signals. Conversely, a relative lack of connectivity during unrewarded behaviour would be expected to lessen the impact of an associated action plan in dlPFC” (page 7752). Thus, the disrupted functional coupling between striatum and orbitofrontal cortex in our case could be a mechanism underlying deficits in reward guided decision-making as often found pathological gambling (van Holst et al., 2010). Confirmation of how altered functional connectivity between frontal and striatal regions affects decision-making performance is an important target for future research.

In contrast to the negative correlations with gambling severity, we found a significant positive correlation in the ‘illusion of control’ contrast between gambling severity and connectivity between the ventral striatum seed and right insula. The same relationship was observed contralaterally at subthreshold significance. These effects were driven by positive striatal-insula correlations with gambling severity on the participant-chosen trials. These data are congruent with accumulating evidence for insula involvement in addiction-related drive states including drug craving (Tang et al., 2012; Verdejo-Garcia et al., 2012). An fMRI study investigating cue-reactivity in pathological gamblers found enhanced insula activity associated with stronger craving to gambling images (Goudriaan et al., 2010). Brain-injury patients with insula damage reported a cessation of cigarette craving compared to patients with damage to other regions (Naqvi et al., 2007), and a similar neuropsychological study using the two reel slot machine task also showed an abolition of the near-miss effect in patients with insula damage (Clark et al., 2014). Given its well-recognized role in the processing of bodily feedback (Craig, 2002), the insula's involvement in addictive behaviours may be to signal the interoceptive aspects of compulsive urges (Gray and Critchley, 2007). Based on the present findings, we would hypothesize that excessive insula recruitment during illusion of control may be a risk factor for the cognitive distortions and loss-chasing that are characteristic of problem gambling.

Our selection of seed regions for the gPPI analyses was based on overall group activation for the contrast of wins versus non-wins; this data-driven approach resulted in seeds that were not bilaterally symmetrical. Our right striatal seed centred on a peak corresponding to nucleus accumbens in the ventral striatum, whereas the left striatal seed region corresponded to the dorsal medial striatum. These hotspots may tap functionally segregated corticostriatal loops (Alexander et al., 1986; Lawrence et al., 1998; Middleton and Strick, 2000; Postuma and Dagher, 2006; Haber and Calzavara, 2009), such that the ventral striatum is particularly implicated in reward-related processing, demonstrating sensitivity to changes in subjective value (Knutson et al., 2001; Rangel et al., 2008) and prediction-based learning (O'Doherty et al., 2004), whereas the dorsal striatum is implicated in action-contingency processing (Delgado, 2007), goal-directed learning (Voorn et al., 2004), instrumental conditioning (O'Doherty et al., 2004) and habit formation (Yin and Knowlton, 2006). Not surprisingly, these striatum subdivisions are also distinguished with respect to cortical connectivity, with the dorsal striatum connected to an associative network with the prefrontal, sensorimotor and parietal association cortices, and the ventral striatum connected with ventral portions of the frontal lobe (Alexander et al., 1986; Middleton and Strick, 2000; Yin and Knowlton, 2006; Haber and Knutson, 2010; Kahnt et al., 2012). Based on this prior knowledge, it is surprising that our connectivity findings for win outcomes were strongest between the dorsal medial striatum seed and the OFC, whereas one would have perhaps expected to find connectivity between the ventral striatal seed and these regions. However, multiple processes during win outcome processing are likely to occur; involving subjective value and prediction-based learning, but also goal-directed learning. Moreover, there is data suggesting that dopamine might direct information flow from ventromedial frontostriatal circuits, implicated in reward and motivation, to more dorsal frontostriatal circuits, associated with cognition and action (Voorn et al., 2004; Haber and Knutson, 2010), this information flow could have been reflected in our results.

Some limitations of the current study should be noted. First, we did not replicate the finding that following the near-misses compared to the (objectively equivalent) full-misses elevated the desire to play the game (Camara et al., 2009; Clark et al., 2012). This was probably due to intermittent nature of ratings in the fMRI version compared to the previous robust findings of behavioural studies outside the scanner. Second, we did not have adequate power to compare near misses either side of the payline, which may have some notable differences (Clark et al., 2013). Third, in the current task, wins, near-misses and full-misses occurred at different probabilities, and thus BOLD differences may also relate to ‘unexpectedness’ (see also Shao et al., 2013; Dymond et al., 2014). During real-life gambling, different gambling outcomes are also not evenly distributed and thus entwined with different unexpectedness. Fourth, the gPPI approach cannot be used to make inferences about directionality. Therefore, identified patterns of connectivity must be grounded in the context of the known neuroanatomy. Fifth, in this study multiple tests were conducted which raises the chance of false positives. Sixth, we used the SOGS questionnaire which is based on DSM-III criteria for pathological gambling (Lesieur and Blume, 1987). Future studies could benefit from using the newer Canadian Problem Gambling Index (CPGI) questionnaire (Ferris and Wynne, 2001). Seventh, we covaried for age and gender, but as our group was predominantly male further studies are required to test whether our effects generalize to female gamblers.

## 5 Conclusions

To conclude, the present data indicate that near-misses are related to a functional network associated with reward processing and learning. More severe gambling problems were associated with lower connectivity between reward-sensitive areas, consistent with previous findings of diminished reward sensitivity in pathological gambling. Furthermore, the connectivity underlying the ‘illusion of control’ effect was stronger within a network associated with craving and bodily arousal in more severe gamblers, which could stimulate gambling behaviour.
